# Uncoupling protein 2 G(-866)A polymorphism: a new gene polymorphism associated with C-reactive protein in type 2 diabetic patients C-reactive protein in type 2 diabetic patients

**DOI:** 10.1186/1475-2840-9-68

**Published:** 2010-10-28

**Authors:** Emanuela Lapice, Michele Pinelli, Elisabetta Pisu, Antonella Monticelli, Roberto Gambino, Gianfranco Pagano, Silvia Valsecchi, Sergio Cocozza, Gabriele Riccardi, Olga Vaccaro

**Affiliations:** 1 Department of Clinical and Experimental Medicine, University of Naples Federico II, via Pansini 5, Napoli, Italy; 2 Department of Cellular and Molecular Biology and Pathology A. Califano DBPCM, University of Naples Federico II, via Pansini 5, Napoli, Italy; 3 Department of Internal Medicine, University of Turin, Corso Achille Mario Dogliotti, 14, Torino, Italy

## Abstract

**Background:**

This study evaluated the relationship between the G(-866)A polymorphism of the uncoupling protein 2 (UCP2) gene and high-sensitivity C reactive protein (hs-CRP) plasma levels in diabetic patients.

**Methods:**

We studied 383 unrelated people with type 2 diabetes aged 40-70 years. Anthropometry, fasting lipids, glucose, HbA1c, and hs-CRP were measured. Participants were genotyped for the G (-866)A polymorphism of the uncoupling protein 2 gene.

**Results:**

Hs-CRP (mg/L) increased progressively across the three genotype groups AA, AG, or GG, being respectively 3.0 ± 3.2, 3.6 ± 5.0, and 4.8 ± 5.3 (p for trend = 0.03). Since hs-CRP values were not significantly different between AA and AG genotype, these two groups were pooled for further analyses. Compared to participants with the AA/AG genotypes, homozygotes for the G allele (GG genotype) had significantly higher hs-CRP levels (4.8 ± 5.3 vs 3.5 ± 4.7 mg/L, p = 0.01) and a larger proportion (53.9% vs 46.1%, p = 0.013) of elevated hs-CRP (> 2 mg/L). This was not explained by major confounders such as age, gender, BMI, waist circumference, HbA1c, smoking, or medications use which were comparable in the two genotype groups.

**Conclusions:**

The study shows for the first time, in type 2 diabetic patients, a significant association of hs-CRP levels with the G(-866)A polymorphism of UCP2 beyond the effect of major confounders.

## Background

Inflammation plays a key role in the pathogenesis of atherosclerosis at every stage, from initiation to progression and rupture of the atherosclerotic plaque [1]. C Reactive Protein (CRP), an acute phase protein synthesized primarily by the liver, is the most widely used biomarker of inflammation and is also a powerful risk marker for cardiovascular diseases [2]. Although obesity, particularly of the visceral type, is a key determinant of hs-CRP levels [3,4], twin and family studies indicate that 35-50% of the phenotypic variation of hs-CRP is genetically determined [5,6]. Uncoupling protein 2 (UCP2) uncouples the substrate oxidation from ATP synthesis, thus decreasing ATP production by the mitochondrial respiratory chain. UCP2 is expressed in spleen, lung, macrophages, and T cells, which suggests a role for UCP2 in the regulation of immunity and inflammatory responsiveness [7]. Furthermore, UCP2 functions as a down-regulator of reactive oxygen species (ROS) whose increased production induces vascular endothelial damage, thus initiating the atherosclerotic process [8,9]. The UCP2 G(-866)A polymorphism is a functional polymorphism located in the promoter region of the gene. In most, although not all studies, the G allele is associated with lower mRNA expression levels [10-14], suggesting a reduced protection against ROS production. In line with this hypothesis, in UCP2 deficient mice the ROS production in macrophages is 80% higher than in wild type mice [15]. In addition, the -866 G allele is associated with an increased risk of chronic inflammatory diseases [16], and greater susceptibility to autoimmune diseases [10] and cardiovascular diseases [17-19].

The aim of this study was to explore the relationship between the G(-866)A polymorphism of UCP2 and hs-CRP plasma levels in type 2 diabetic patients. The role of major confounders was also studied. This association has never been explored, although potentially relevant since it may contribute to the understanding of the possible mechanisms linking this polymorphism to cardiovascular risk.

## Methods

Population: The study population was recruited at two centres in two Italian cities (Naples and Turin), within the framework of a project aimed at studying the emerging risk factors for the chronic complications of diabetes, partially funded by the Italian Ministry of University, Research and Technology (MURST prot. 2004-062128-001). Study participants were 383 (189 men and 194 women) unrelated patients with type 2 diabetes mellitus consecutively seen at the outpatient clinics and recruited according to the following criteria: type 2 diabetes diagnosed according to WHO, and aged 40 to 70 years. Exclusion criteria were overt renal or hepatic diseases (serum creatinine > 2 mg/dl, ALT > three times the normal values), autoimmune disorders, chronic use of steroids or non steroidal anti-inflammatory drugs, statin therapy, acute or chronic infections (assessed by history, use of drugs, clinical examination, and white cell count > 10.000/μL). The study was approved by the local ethics committee and was conducted in accordance to the Helsinki Declaration. All participants signed an informed consent. The study was conducted according to a standard protocol by ad-hoc trained investigators who were unaware of the participant's genotype status. All investigations took place in the morning.

### Measurements

Weight, height, and waist circumference were measured with patients in light clothing and without shoes. BMI was calculated. Waist circumference was measured at the level of the umbilicus. Medication use was assessed by interview and validated against clinical charts on a random sample. Fasting glucose, triglycerides, total and HDL cholesterol were measured by standard laboratory methods on fresh plasma. Glycated hemoglobin was measured by HPLC. Laboratory measurements were standardized in the two centres. For all participants, CRP was measured in Torino by a high sensitivity method (Tina-Quant, Roche/Hitachi 904) on frozen samples of EDTA-treated plasma.

### Genotyping

Genotype analyses were centralized in Naples. Genomic DNA was isolated from whole blood using Biorobot EZ1 Qiagen. All the patients were genotyped for the G(-866)A (rs659399) variation in the promoter region of Uncoupling Protein 2 (UCP2) by polymerase chain reaction (PCR). Oligoprimers were tested by gradient PCR to optimize melting temperature. Genotyping was performed by an allele-specific amplification method using SYBR Green detection in a Realtime ABI PRISM 7000 apparatus (PE Applied Biosystem). To ensure genotyping accuracy, the laboratory received 5% of the samples in blind duplicates and results were fully concordant. The G(-866)A genotype distribution was in Hardy-Weinberg equilibrium and similar in the two centres.

## Statistical analysis

Data is given as means and standard deviations or percentages. Skewed variables (i.e. plasma triglycerides, insulin and hs-CRP) were log transformed for the analyses and back transformed to the original values in tables and figures. The X^2 ^goodness-of-fit test was used to assess deviation from Hardy-Weinberg equilibrium of the genotypic frequency by calculating expected frequencies of genotypes. Group means were compared by unpaired Student's t-test or analysis of variance, as appropriate. Proportions were compared by X^2 ^square analysis. Multivariate analysis was conducted by linear regression with hs-CRP as the outcome variable, the independent variables included in the model were G(-866)A polymorphism of UCP2, gender, age, glycated hemoglobin, waist circumference, centre (Napoli - Torino). A p-value of less than 0.05 (two tailed) was considered significant. All statistical analyses were conducted using Statistical Package for Social Science (SPSS) for Windows version 14.0

## Results

The study participants were middle aged (58.1 ± 7.9 years) and generally overweight (BMI 30.7 ± 5.9 kg/m^2^). Glucose control, as assessed by HbA1c, was on average not optimal (HbA1c 7.4 ± 1.7%). The UCP2 genotype distribution was in Hardy Weinberg equilibrium and similar to that observed in other Caucasian populations: i.e. 10.2% of the participants were AA, 41.5% were AG and 48.3% were GG.

**Figure 1:**
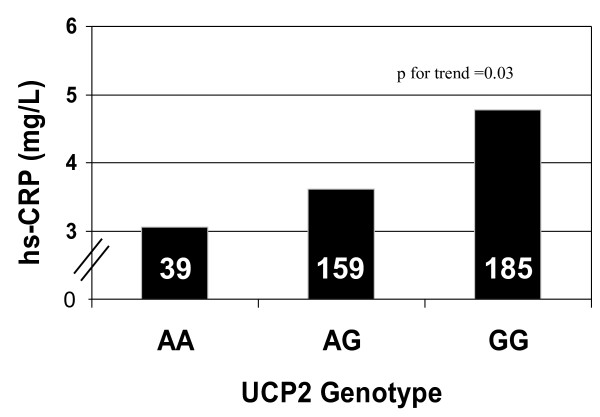
**CRP plasma concentrations according to UCP2 genotype (p = 0.03 for trend)**.

Hs-CRP plasma concentrations are shown in figure 1 by UCP2 genotype. Values progressively increased across the three genotype groups, being lowest for carriers of the AA genotype, intermediate for those with the AG genotype and highest for the GG genotype, (respectively 3.0 ± 3.2 vs 3.6 ± 5.0 vs 4.8 ± 5.3 mg/L; p = 0.03, for linear trend), thus suggesting a gene dose effect. The ANOVA analysis of variance with post hoc tests showed a statistically significant difference in hs-CRP values between the GG genotype participants and those with the AG (p < 0.02) or the AA genotype (p = 0.05) (figure 1). Due to the small number of people with the AA genotype, this group was pooled together with the AG genotype in subsequent analyses.

Hs-CRP levels were 4.8 ± 5.3 vs 3.5 ± 4.7 mg/l in carriers of the GG genotype compared to those with the AA/AG genotype (p = 0.01); accordingly, the proportion of people with hsCRP > 2 mg/L - a value associated with increased cardiovascular risk [20] - was significantly higher in the group with the GG genotype (53.9% vs 46.1%; p = 0.013) (table 1). No significant difference between the two groups was observed for BMI and waist circumference, two key determinants of CRP. Age, gender, HbA1c, lipids and smoking status were also comparable in the two genotype groups as it was the proportion of patients treated with insulin, insulin secretagogues, or insulin sensitizers (table 1).

**Table 1:** Characteristics of the study participants by UCP2 genotype

UCP 2 G(-866)A polymorphism	AA+AG (N 198)	GG (N 185)	p
Age (years)	58.2 ± 7.5	58.0 ± 8.2	0.78

Males (%)	52.5	47.5	0.22

BMI (kg/m^2^)	30.5 ± 5.9	30.9 ± 5.8	0.46

Waist circumference (cm)	100.9 ± 11.9	101.2 ± 12.0	0.82

Total cholesterol (mg/dl)	197.9 ± 37.9	205.2 ± 43.9	0.09

HDL cholesterol (mg/dl)	48.4 ± 11.7	48.6 ± 12.4	0.93

Triglycerides (mg/dl)	149.8 ± 100.8	146.8 ± 91.8	0.75

HBA1c (%)	7.4 ± 1.7	7.4 ± 1.7	0.78

Hs-CRP (mg/l)	3.5 ± 4.7	4.8 ± 5.3	0.01

Proportion (%) with hs-CRP > 2 mg/L	46.1	53.9	0.01

Current smokers (%)	47.0	36.8	0.10

Metformin (%)	25.6	29.7	0.48

Insulin secretagogues	19.8	25.0	0.61

Insulin (%)	54.5	45.3	0.34

Multivariate regression analysis (Table 2) confirmed a significant association of the G(-866)A polymorphism of UCP2 with hs-CRP independent of gender, age, waist circumference, glucose control and centre. The finding remained very much the same when a different model was used, with BMI instead of waist circumference as a covariate. A significant association of CRP with gender and waist circumference was also observed.

**Table 2:** Independent association of the UCP2 genotype and relevant phenotypic parameters with plasma hs-CRP levels

	Dependent variable: hs-CRP		
**Independent variables**	B	Se B	p

**Age (years)**	0.001	0.001	0.291

**Gender (M%)**	0.031	0.012	0.008

**Waist circumference (cm)**	0.003	0.001	0.001

**HbA1c (%)**	0.006	0.003	0.083

**UCP2 AG-GG/GG**	0.024	0.012	0.037

**Centre (Naples/Turin)**	-0.008	0.013	0.540

## Discussion

The results of this study show that in people with type 2 diabetes the G allele of the UCP2 G(-866)A polymorphism is associated with elevated hs-CRP levels with a gene dose effect, thus suggesting a markedly higher inflammatory status in carriers of the GG genotype compared to carriers of the AA/AG genotype. The finding holds after correction for confounders such as waist circumference, BMI and blood glucose control. In addition, people with acute or chronic inflammatory conditions were excluded from the study thus ruling out further confounders. The precise function of UCP2 is still unknown. However, based on available evidence, the UCP2 gene is a plausible biological candidate for a role in the regulation of the inflammatory response. The gene is expressed in a variety of cells including spleen cells, macrophages and T cells. Moreover, the UCP2 protein functions as a down-regulator of reactive oxygen species (ROS) generation [15]. Several studies have shown that the UCP2 G(-866)A is a functional polymorphism with the G allele associated with lower mRNA expression levels [10-14]. This is compatible with a reduced protection against ROS production [21]. ROS modulate multiple signal transduction pathways - activation of matrix metalloproteinase, vascular smooth muscle cell proliferation and death, endothelial dysfunction and lipid peroxidation - all of which are implicated in the process of atherogenesis [22]. In addition, ROS can activate the transcription factor NF-kB, that is ubiquitously expressed and mediates the expression of genes involved in the acute inflammatory response, including the cytokines IL1-IL6 and IL8 [23], which in turn modulate CRP production. Therefore, it is plausible to hypothesize that functional UCP2 gene polymorphisms leading to reduced expression of UCP2 may be related to a proinflammatory status and possibly to the development and progression of atherosclerosis. This hypothesis is supported by studies in animals and in cell culture [24,25]. Transplantation of UCP2 deficient bone marrow cells to low density lipoprotein receptor deficient mice markedly accelerated the formation of atherosclerotic lesions. Moreover, arterial walls isolated from these mice had increased macrophage accumulation in the atherosclerotic lesions [24].

In humans the common G allele was associated with lower mRNA expression levels in several studies [10-14]. The G allele has also been associated with an increased risk of chronic inflammatory diseases [10,16] and cardiovascular diseases [17]. Our study expands current knowledge by providing the evidence that the GG genotype of UCP2 is associated with increased circulating hs-CRP levels beyond the effect of major confounders. To our knowledge, while there is no previous data reporting a relationship between the G(-866)A polymorphism of UCP 2 and hs-CRP which hampers comparison, the results of the present study are in line with two recent observational studies. One, conducted in healthy children and adolescents, showed that the G variant of UCP2 was associated with markers of low grade inflammation (i.e. fibrinogen, complement C3 and complement C4) [26]. The other [17] demonstrated in two large independent cohorts of diabetic subjects that the G allele was associated with increased risk of coronary artery disease compared to the A allele, independent of major CV risk factors; unfortunately CRP was not measured. However, the association between the G(-866)A polymorphism of the UCP2 and cardiovascular diseases is still debated, as it is often the case with genetic association studies, since some studies showed a protective effect of the A allele against cardiovascular diseases [18,19], which is somewhat at variance with our results.

The role of genes in the etiology of cardiovascular diseases and diabetes needs to be further explored. These conditions are rapidly increasing worldwide largely due to rapid changes in lifestyle related factor. However, the genetic background plays a role in determining individual susceptibility in response to similar environmental conditions [27-29] and may provide a basis for identifying at risk individuals at a young age thus allowing early prevention.

Among the study limitations we must acknowledge that, due to the cross-sectional design, we cannot determine whether the UCP2 polymorphism is causally associated with hs-CPR or the association is mediated through other atherosclerosis-associated conditions. A further limitation is the fact that hs-CRP levels were measured only once in this study. However it has been shown that the intraindividual variation of hs-CRP is low and that this parameter is reasonably stable when compared at various points in time, several years apart [30]. In addition, we carefully excluded people with acute or chronic inflammatory conditions thus further reducing variability.

## Conclusion

This study provides the evidence of an association between UCP2 G(-866)A polymorphism and hs-CRP levels in type 2 diabetic patients. The finding may contribute to the understanding of the role of this polymorphism in the modulation of cardiovascular risk by providing evidence for a biologically plausible mechanism. This being the first report, the findings need to be replicated in other populations with or without diabetes.

## Competing interests

The authors declare that they have no competing interests.

## Author contributions

EL: Researched data, Wrote manuscript. MP: Researched data.

EP: Researched data. AM: Reviewed/edited manuscript. RG: Researched data.

GP: Reviewed/edited manuscript. SV ' deceased': Researched data. SC: Reviewed/edited manuscript. GR: Contributed to discussion, Reviewed/edited manuscript.

OV: Wrote manuscript, Contributed to discussion, Reviewed/edited manuscript

All authors read and approved the final manuscript.
